# Pangenome-scale analysis of the PYL gene family reveals evolutionary conservation, regulatory diversification and salt-responsive haplotypes in *Brassica napus*

**DOI:** 10.3389/fpls.2026.1870359

**Published:** 2026-06-08

**Authors:** Xin Liu, Yongxin Nie, Xuyang Chen, Xingge Ding, Tingting Chen, Yuanlin Zhao, Xin Zhao, Yue Yan, Minghu Zhang, Meng Liu

**Affiliations:** 1 Faculty of Agriculture, Forestry and Food Engineering, Yibin University, Yibin, Sichuan, China; 2 Oil Crops Research Institute, Yibin Academy of Agricultural Sciences, Yibin, Sichuan, China; 3 State Key Laboratory of Crop Gene Exploration and Utilization in Southwest China, Sichuan Agricultural University, Chengdu, Sichuan, China; 4 School of Science, Xi’an Jiaotong-Liverpool University, Suzhou, China

**Keywords:** *Brassica napus*, haplotype variation, pangenome, PYL gene family, salt stress

## Abstract

Abscisic acid (ABA) signaling is a central determinant of plant stress adaptation, yet the evolutionary and functional diversity of its receptor family remains poorly understood at the pangenome scale in polyploid crops. We analyzed the PYL ABA receptor family across eight high-quality *Brassica napus* genomes and identified 405 genes, revealing a predominantly conserved core repertoire together with a limited but dynamic dispensable fraction. Although *BnPYLs* retained a highly conserved phylogenetic framework, gene gain and loss varied among clades, and structural diversification was concentrated in non-core members. Promoter and transposable-element analyses indicated that regulatory-region variation may have contributed to family diversification, with the presence of putative MYB/MYC, ABA- jasmonate, and light-responsive cis-elements and widespread transposable-element accumulation in flanking regions. Copy number, structural variation and duplication analyses showed that the family was shaped primarily by ancient whole-genome duplication, followed by localized copy-number changes and asymmetric structural remodeling between subgenomes. Despite this genomic plasticity, *BnPYLs* remained under pervasive purifying selection and displayed strong syntenic conservation across accessions. Expression profiling uncovered substantial transcriptional divergence, including widespread root-preferential expression and selective induction of specific *BnPYLs* under salt stress. Four salt-responsive genes were validated by qRT–PCR, and haplotype analysis further showed that natural allelic variation at these loci is associated with yield performance under saline–alkaline conditions. These findings establish a pangenome framework for understanding the evolutionary stability and functional diversification of ABA receptors in rapeseed, and provide candidate allelic resources for future functional validation and breeding improvement of stress resilience and yield stability.

## Introduction

Abscisic acid, or ABA, is a central plant hormone that regulates seed dormancy, germination, root growth, stomatal movement, senescence, and adaptation to drought, salinity, and temperature stress (Zhai et al., 2025). A key advance in ABA biology was the identification of the PYRABACTIN RESISTANCE 1, PYR1 LIKE, and REGULATORY COMPONENT OF ABA RECEPTOR family, collectively referred to as PYR/PYL/RCAR, as the core ABA receptors that initiate the canonical ABA signaling pathway (Fujii et al., 2009; Ma et al., 2009). Upon ABA binding, PYL proteins inhibit clade A PP2Cs and thereby release SnRK2 kinases, which activate downstream transcription factors and stress responsive genes (Cutler et al., 2010). Because this receptor module sits at the entry point of ABA signaling, PYL genes are central to the coordination of plant development and environmental adaptation.

Studies in *Arabidopsis* and crop species have shown that the PYL family is conserved in biochemical function but diversified in gene number, duplication history, expression pattern, and stress responsiveness (Antoni et al., 2013; Kim et al., 2014; Verma et al., 2019). In rice, pear, tea, and eggplant, genome-wide analyses consistently showed that PYL genes are usually retained under purifying selection, often expanded through whole genome or segmental duplication, and associated with both tissue development and abiotic stress responses (Yadav et al., 2020; Wang et al., 2022; An et al., 2023). Functional studies have further shown that different receptors can play different biological roles. For example, *AtPYL8* is important for root ABA signaling and lateral root growth, *AtPYL9* promotes drought resistance and leaf senescence related responses, and engineered or selected PYL alleles in rice and wheat can improve drought or cold tolerance, although strong activation of ABA signaling may also reduce growth (Zhao et al., 2014) These findings suggest that the PYL family follows a common evolutionary pattern in plants: a conserved signaling core is maintained, while individual paralogues diverge in regulation and physiological output.

*Brassica napus* is a globally important allotetraploid oilseed crop that contributes about 13 to 16% of vegetable oil production worldwide (Nesi et al., 2008). It originated from hybridization between *B. rapa* and *B. oleracea* and later differentiated into winter, semi winter, and spring ecotypes adapted to different environments (Tan et al., 2024). Owing to its recent polyploid origin and broad ecotype diversification, the rapeseed genome contains abundant copy number variation, presence and absence variation, and structural variation (Zhang et al., 2024; Afsharyan et al., 2025). An earlier reference based study identified 46 PYL genes in *B. napus* and showed that several members respond to drought, heat, and salinity stress (Di et al., 2018). However, in a species with extensive intraspecific genomic variation, a single reference genome cannot fully represent the complete gene repertoire of a regulatory family, especially one that may be shaped by duplication, gene loss, and structural change across accessions.

This limitation makes a pangenome framework especially important for gene family analysis. A pangenome captures the full gene space of a species and distinguishes core genes shared across genomes from dispensable genes that vary among lineages (Golicz et al., 2016). In plants, pangenome analysis has become a powerful approach for identifying presence and absence variation, copy number variation, and structural variants that are often missed by single reference studies (Danilevicz et al., 2020). In rapeseed, the availability of eight high quality genomes has already shown that core and dispensable gene sets, large scale structural variants, and ecotype specific genomic changes contribute substantially to phenotypic diversity (Song et al., 2020). More recent pangenomic work further demonstrated that structural variation reshapes gene expression and trait variation across large rapeseed populations (Zhang et al., 2024; Afsharyan et al., 2025). For a signaling family such as PYL, this approach has clear advantages. It allows more accurate estimation of family size, classification of core and non- core members, detection of subgenome biased gain and loss, and direct evaluation of how structural and regulatory variation may shape tissue expression, stress response, and agronomic traits.

Against this background, a pangenome scale analysis of the *B. napus* PYL family is needed to understand how ABA receptors have evolved in a recently formed polyploid crop. Such an analysis can move beyond simple gene cataloguing and provide a population level view of family conservation, gene structure, promoter composition, transposable element association, copy number change, structural variation, expression divergence, stress responsive candidates, and useful haplotypes. In the present study, the PYL gene family was systematically analyzed across eight *B. napus* genomes, with the aim of resolving its conserved and variable components and identifying loci with potential value for stress adaptation and rapeseed improvement.

## Materials and methods

### Identification of *BnPYLs*

To identify PYL gene family members in *Brassica napus*, genome assembly files, gene annotation files, and annotated protein datasets for eight rapeseed genomes, including ZS11.v0, GanganF73.v0, No2127.v0, QuintaA.v0, Shengli3.v0, Tapidor.v0, Westar.v0, and Zheyou73.v0, were downloaded from the genomics module of the download section in the BnIR database (https://yanglab.hzau.edu.cn/BnIR/download?module=genomics). The Hidden Markov Model (HMM) profile corresponding to the conserved PYL domain (Pfam accession PF10604) was retrieved from the Pfam database and used as the query to perform HMM searches against the protein sets of the eight genomes using HMMER v3.0 (Mistry et al., 2013). Candidate sequences were screened with an appropriate E-value threshold, and redundant sequences or incomplete protein models were removed. The protein sequences, CDS, and genomic sequences of the retained candidates were then extracted from the corresponding genome assemblies.

To ensure the reliability of gene family identification, all candidate proteins were further examined for the presence of the conserved PYL domain using multiple domain annotation databases, including the CDD (https://www.ncbi.nlm.nih.gov/cdd/), SMART (http://smart.embl-heidelberg.de/), and Pfam (http://pfam.xfam.org/). Only proteins containing the intact conserved domain were retained as authentic *BnPYLs*.

### Pangenome-based classification of *BnPYLs*

To evaluate the conservation and variation patterns of the PYL family across the rapeseed pangenome, orthologous gene groups were inferred using OrthoFinder (Emms and Kelly, 2019). According to their distribution across the eight accessions, these orthologous groups were classified into four categories: core groups, present in all eight genomes; soft-core groups, detected in seven genomes; shell groups, present in two to six genomes; and line_specific/cloud groups, found in only one genome. This classification enabled the identification of conserved and variable PYL members and facilitated subsequent analyses of copy number variation, lineage-specific gain or loss, and subgenome-biased evolutionary patterns. Multiple copies within the same genome were treated as copy-number variation within the corresponding orthogroup. For pangenome classification, an orthogroup was therefore considered present in a genome if at least one gene copy was detected, regardless of copy number. The number of gene copies assigned to each orthogroup in each genome was recorded separately and used for copy-number analysis.

### Phylogenetic analysis of *BnPYLs*

To investigate the evolutionary relationships of the PYL gene family, a phylogenetic tree was constructed using full-length PYL protein sequences from *Brassica napus* and *Arabidopsis* thaliana. Protein sequences were aligned with MUSCLE, and ambiguously aligned positions were subsequently filtered using trimAl to improve the robustness of downstream analyses. The refined alignment was then subjected to phylogenetic reconstruction under the Maximum Likelihood (ML) framework in IQ-TREE v2.0 (Nguyen et al., 2015). The best-fit substitution model was automatically selected according to the program’s built-in model test. Branch support was evaluated using 1,000 bootstrap replicates, and the final tree file was visualized and annotated with the Interactive Tree of Life (iTOL) online platform (http://itol.embl.de/).

### Gene structure, conserved motif, and promoter cis-element analyses of *BnPYLs*

Structural and regulatory features of *BnPYLs* were further characterized at multiple levels. Exon–intron organization was obtained from the annotation files of the eight *B. napus* genomes. Conserved motifs in BnPYL proteins were identified using the MEME tool in the MEME Suite. Full-length amino acid sequences of all identified BnPYL proteins were used as input. MEME was run with the zero-or-one occurrence per sequence model, the maximum number of motifs was set to 10, and the motif width was restricted to 6–50 amino acids. The maximum E-value was set to 10, and other parameters were kept as default. The gene structure and motif composition of each BnPYL member were then integrated and displayed using TBtools (Chen et al., 2023). To explore the potential transcriptional regulation of *BnPYLs*, the 2-kb genomic region upstream of each gene start codon was extracted as the putative promoter sequence. These promoter regions were scanned for cis-acting regulatory elements using the PlantCARE database (Lescot et al., 2002). Identified elements were grouped according to their predicted functions, including hormone responsiveness, abiotic stress responsiveness, light responsiveness, and growth- or development-related regulation.

### TE insertion analysis of *BnPYLs*

TE annotation files were obtained from the Supporting Information datasets of the rapeseed Pan-TE map study published by Yang et al. (2026). The TE annotation datasets are publicly available through Zenodo (https://doi.org/10.5281/zenodo.16959683). For each genome, TE annotations in GFF3 format were used to identify TE insertions associated with BnPYL loci. The genomic coordinates of *BnPYLs* were compared with TE annotation coordinates using custom Python scripts. For each BnPYL locus, three regions were defined: the gene body, a 2-kb upstream region, and a 2-kb downstream region. TE annotations overlapping these regions were extracted and classified as upstream, genic, or downstream according to their relative positions. For UTRs, when included in the gene annotation, were counted as part of the genic region, but UTRs were not analyzed as an independent category.

### Synteny and Ka/Ks analyses of *BnPYLs*

To investigate the evolutionary conservation and divergence of *BnPYLs*, synteny analysis was first performed across eight Brassica napus genomes using MCScanX (Wang et al., 2012). Briefly, protein sequences were compared using an all-against-all Diamond search, and the resulting similarity file together with gene position files was used as input for MCScanX. Collinear blocks containing PYL loci were identified, and homologous gene pairs associated with *BnPYLs* were extracted to evaluate the positional conservation of this gene family across different rapeseed accessions. In the pairwise comparisons between ZS11 and each of the other seven genomes, a PYL gene was considered collinear when a ZS11 PYL and its homologous PYL counterpart in the compared genome were included in the same collinear block. The identified collinear relationships were then visualized to compare the genomic organization of *BnPYLs* among the eight genomes. Duplication types of *BnPYLs* were classified using the duplicate gene classification module in MCScanX. Genes located within collinear blocks were classified as whole-genome duplication or segmental duplication genes. Duplicated genes located next to each other on the same chromosome were classified as tandem duplicates, whereas duplicated genes located close to each other but not immediately adjacent were classified as proximal duplicates, following the MCScanX classification scheme. Duplicated genes that were not assigned to the whole-genome/segmental, tandem, or proximal categories were classified as dispersed duplicates.

Based on the orthologous or collinear PYL gene pairs, the numbers of nonsynonymous substitutions (Ka) and synonymous substitutions (Ks) were calculated using the Simple Ka/Ks Calculator (Wang et al., 2010). The resulting Ka/Ks ratios were used to estimate the selective pressure acting on *BnPYLs* during evolution. In general, a Ka/Ks ratio of less than 1 indicates purifying selection, a ratio of 1 suggests neutral evolution, and a ratio greater than 1 reflects positive selection. To illustrate the overall distribution of evolutionary constraint across the PYL family, the Ka/Ks values were summarized and visualized in R using the ggplot2 and ggridges packages.

### Structural variation and synteny analysis

Structural variations affecting PYL loci were identified by comparing the ZS11 genome with the other seven *Brassica napus* genomes in the pangenome dataset. Whole-genome pairwise alignments were generated using minimap2 (Li, 2018), alignment results were output in PAF format. INS/DEL candidates were inferred by comparing query–reference coordinate gaps between adjacent collinear alignment blocks. For each pair of adjacent blocks on the same chromosome and strand, the unaligned intervals in the ZS11 reference and query genomes were calculated. A larger query gap was classified as an insertion relative to ZS11, whereas a larger reference gap was classified as a deletion. Event size was defined as the absolute difference between the two gaps. Alignments were filtered using mapping quality ≥5 and minimum alignment length ≥50,000 bp for variant calling, and INS/DEL events of 20–1,000,000 bp were retained.

To identify SVs associated with BnPYL loci, the genomic region of each BnPYL gene was extended by 2 kb upstream and downstream. INS/DEL events overlapping the gene body or its ±2kb flanking region were retained using a custom Python script.

### Codon usage evaluation

To characterize codon usage features of the BnPYL gene family, the coding sequences of *BnPYLs* were subjected to codon bias analysis using CodonW v1.4.2. Several commonly used indices were calculated, including the effective number of codons (ENC), codon adaptation index (CAI), relative synonymous codon usage (RSCU), overall GC content, GC3s, and base frequencies at the synonymous third codon position (A3s, T3s, G3s, and C3s). These parameters were used to evaluate the extent and compositional features of codon preference among *BnPYLs*.

To further explore the forces shaping codon usage in *BnPYLs*, PR2 bias analysis was performed based on nucleotide frequencies at the third codon position. For each gene, the values of A3/(A3 + T3) and G3/(G3 + C3) were calculated and plotted to assess deviations from equal usage of complementary bases. The coordinate (0.5, 0.5) was used as the theoretical equilibrium point, and departures from this point were interpreted as evidence that codon usage was influenced by factors other than simple mutational equilibrium.

In addition, an ENC versus GC3s plot was generated to distinguish the relative effects of nucleotide compositional constraint and selective pressure on synonymous codon choice. Observed ENC values for individual *BnPYLs* were plotted against their corresponding GC3s values and compared with the expected curve under mutation driven codon usage. All figures were generated in Python using Matplotlib (Barrett et al., 2005), and the same plotting scheme was applied across all analyzed *BnPYLs* to facilitate direct comparison.

### Expression profiling and qRT-PCR validation of *BnPYLs*

To characterize the transcriptional patterns of *BnPYLs*, expression datasets were retrieved from the BnIR database (ZS11 expression atlas) (https://yanglab.hzau.edu.cn/BnIR/expression_zs11). Basal transcript abundance of *BnPYLs* was collected from nine representative tissues, including bud, flower, leaf, seed, silique, root, cotyledon, rosette, and stem. In addition, time course expression data under salt stress were obtained from root and leaf tissues at 0.25, 0.5, 1, 3, 6, 12, and 24 h after treatment. The expression values provided by BnIR were log2(value+1)-transformed before visualization. For heatmap generation, z-score normalization was performed by row, meaning that each BnPYL gene was standardized across the analyzed tissues or treatment time points. This approach was used to display the relative expression pattern of each gene. Heatmaps were generated using the ComplexHeatmap package in R (Gu et al., 2016).

To further validate the salt responsive expression of selected *BnPYLs*, seedlings of the *Brassica napus* cultivar Chuanyou 83 were grown under controlled environmental conditions and treated with 200 mM NaCl at the seedling stage. Root samples were collected at 1, 3, 6, 12, and 24 h after treatment, whereas untreated seedlings served as controls. For each treatment and time point, three independent biological replicates were collected, and each biological replicate consisted of pooled root tissues from multiple seedlings. or each biological replicate, three technical replicates were performed in the qRT-PCR assay. All samples were immediately frozen in liquid nitrogen and stored at −80°C before RNA isolation. Total RNA was extracted using the RNAprep Pure Plant Kit (Tiangen, China), and first strand cDNA was synthesized from 1 μg of total RNA using the RevertAid First Strand cDNA Synthesis Kit (Thermo Scientific, USA). The resulting cDNA was diluted 50-fold with DEPC-treated water before qRT-PCR analysis. Gene-specific primers for the selected *BnPYLs* were designed using Primer 5 (**Supplementary Table 1**). Quantitative real-time PCR was performed using TB Green Premix Ex Taq II (Tli RNaseH Plus; Takara, Japan) in a 10 μL reaction volume containing 5 μL of 2× TB Green Premix Ex Taq II, 0.4 μL of each forward and reverse primer (10 μM), 1 μL of diluted cDNA, and nuclease-free water to the final volume. The qRT-PCR program was as follows: initial denaturation at 95°C for 3 min, followed by 39 cycles of denaturation at 95°C for 10 s and annealing/extension at 58°C for 30 s. After amplification, melting-curve analysis was performed by denaturation at 95°C for 5 s, followed by a gradual increase from 65°C to 95°C at 0.5°C increments. Primer specificity was assessed based on the presence of a single sharp melting peak and the absence of obvious nonspecific amplification or primer-dimer signals. Relative transcript levels were calculated using the 2^^−ΔΔCt^ method (Livak and Schmittgen, 2001), with the actin gene used as the internal reference.

### Haplotype analysis of *BnPYLs* and phenotypic association

To investigate natural allelic variation in the BnPYL family, sequence polymorphism data, including SNPs and InDels, were obtained from the multi locus variation module of the BnIR database. Variant effects were annotated according to the information provided by BnIR, including their positions relative to gene structure, such as CDS and UTR regions, and their predicted impact categories, including low, moderate, and modifier effects. Variants located within candidate *BnPYLs* and their corresponding regulatory regions were extracted and used to define haplotypes. Accessions carrying different haplotypes were then grouped for downstream comparison.

Haplotypes were defined as unique allelic combinations across selected biallelic SNP and InDel sites within each candidate BnPYL gene and its regulatory region. Accessions with identical allele patterns were assigned to the same haplotype group, whereas accessions with heterozygous or missing calls at haplotype-defining sites were excluded from the corresponding gene analysis. This filtering resulted in different retained accession numbers among genes.

Phenotypic data for accessions were downloaded from the phenotype module of the download section in the BnIR (https://yanglab.hzau.edu.cn/BnIR/download?module=genomics), and were based on the field trial dataset reported by Zhang et al. (2022). These data were generated from field experiments conducted under natural saline–alkaline conditions rather than controlled greenhouse treatments. In that study, 505 *Brassica napus* accessions were evaluated in Wuyuan County, Bayannur City, Inner Mongolia, China, located in the Hetao Plain. The experimental soils were divided into three saline–alkaline levels: control soil with salinity of 0.10–0.25% and pH 7.5–8.0, low saline–alkaline soil with salinity of 0.35–0.53% and pH 8.0–8.5, and high saline–alkaline soil with salinity of 0.64–1.05% and pH 8.3–9.0. In the original field experiment, each accession was planted in four rows with 20 cm spacing between rows and between plants, and each row contained eight plants. An additional technical replicate was established in a nearby saline–alkaline field.

In the present study, yield-related traits from the 505 rapeseed accessions grown under saline–alkaline conditions were used to evaluate the potential phenotypic effects of different BnPYL haplotypes. Yield data from the high saline–alkaline condition were used to assess variation associated with haplotype differences. By integrating sequence polymorphism, haplotype classification, and field phenotypic data, this analysis provided a basis for identifying favorable BnPYL alleles potentially associated with salt-alkali tolerance and agronomic performance.

## Results

### Identification and physicochemical characterization of the *BnPYLs* across eight *Brassica napus* genomes

**Figure 1:**
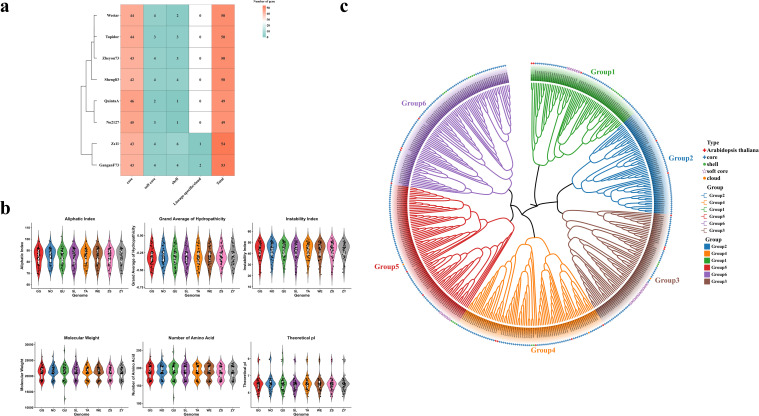
Pangenome-scale characterization of the PYL gene family in *Brassica napus*. **(A)** Classification of *BnPYLs* across eight rapeseed genomes into core, soft-core, shell and line_specific/cloud categories. Numbers in each cell indicate gene counts for each category in each accession, and the total number of *BnPYLs* is shown in the rightmost column. **(B)** Distribution of physicochemical properties of *BnPYLs* across the eight genomes. **(C)** Maximum-likelihood phylogeny of *BnPYLs* and *Arabidopsis thaliana*. The family is resolved into six major clades (Groups 1–6). Tip annotations indicate gene categories in the pangenome, including core, soft-core, shell and line_specific/cloud members, with *A. thaliana* PYLs shown as references.

A total of PYL genes were identified across eight *Brassica napus* genomes, with moderate variation in gene number among accessions (**Figure 1A**; **Supplementary Table 2**). Specifically, 50 PYL genes were detected in Westar, Tapidor, Zheyou73, and Shengli3, whereas 49 genes were identified in QuintaA and No2127. In contrast, slightly higher numbers were observed in ZS11 (54 genes) and GanganF73 (53 genes), indicating moderate expansion of the PYL gene family in specific genomes.

To further characterize these proteins, their physicochemical properties were systematically analyzed (**Supplementary Table 3**; **Figure 1B**). The predicted protein length ranged from approximately 120 to 280 amino acids, corresponding to molecular weights of ~12.7–28.06 kDa. The theoretical isoelectric point (pI) values spanned a broad range (~4.56–9.68), suggesting that PYL proteins may function under diverse intracellular pH conditions. The aliphatic index values were primarily distributed between ~68.59 and 102.41, indicating relatively high thermostability of most *BnPYLs*. The grand average of hydropathicity (GRAVY) ranged from approximately −0.6 to 0.2, with most proteins showing negative values, consistent with a predominantly hydrophilic nature. In addition, the instability index ranged from ~21 to 55, with a substantial proportion of proteins predicted to be stable (instability index < 40), although some members may exhibit reduced stability. Collectively, these results indicate that while the copy number of *BnPYLs* is relatively conserved across the eight genomes, their physicochemical properties exhibit notable diversity, suggesting potential functional differentiation of *BnPYLs*.

### Core and non-core composition of the *BnPYLs* across eight *Brassica napus* genomes

To assess the conservation and dispensability of the PYL gene family in *Brassica napus*, *BnPYLs* identified across eight genomes were classified into core and non-core categories based on their distribution patterns (**Figure 1A**; **Supplementary Table 2**). Overall, core genes represented the largest proportion of the PYL repertoire in all accessions, indicating that most PYL members are highly conserved during *B. napus* evolution. The number of core genes ranged from 43 to 46, with the highest number observed in No2127 (46), followed by Westar, Tapidor, and QuintaA (45 each), whereas Zheyou73, ZS11, and GanganF73 each contained 44 core genes, and Shengli3 harbored the fewest (43).

In contrast, non-core genes showed greater variation among accessions. Soft-core genes ranged from 1 to 5 copies, with one gene detected in Tapidor, QuintaA, and No2127, two in Westar, four in Zheyou73 and Shengli3, and five in both ZS11 and GanganF73. Shell genes were present at 2–4 copies across genomes, including three in Westar, four in Tapidor and ZS11, three in QuintaA and Shengli3, and two in No2127, Zheyou73, and GanganF73. Lineage-specific genes were rare, being absent from six genomes and detected only in ZS11 (one gene) and GanganF73 (two genes). Consistently, total PYL gene numbers ranged from 49 to 54, with the highest counts in ZS11 (54) and GanganF73 (53), and the lowest in QuintaA and No2127 (49 each). These results indicate that the PYL gene family in B. napus is characterized by a predominantly conserved core component together with a relatively small but variable non-core fraction. Such a distribution pattern suggests that most *BnPYLs* may perform essential and conserved biological functions, whereas the dispensable members, especially lineage-specific genes, may have contributed to genomic diversification among different accessions.

### Phylogenetic grouping of *BnPYLs* in *Brassica napus*

To investigate the evolutionary relationships of *BnPYLs*, a phylogenetic tree was constructed using protein sequences of *BnPYLs* from eight *B. napus* genomes, with *Arabidopsis thaliana* PYLs included as references (**Figure 1C**). The resulting tree resolved the *BnPYLs* into six major clades (Groups 1–6), indicating a well-defined and evolutionarily conserved phylogenetic framework.

Despite this overall conservation, the distribution of non-core genes varied markedly among clades, revealing distinct evolutionary dynamics. Group 6, the largest clade (83 genes), was composed almost entirely of core members (76 core and 7 shell genes), with no soft-core or line_specific/cloud genes detected. Group 5 (79 genes) displayed the greatest compositional diversity, comprising 67 core, 7 soft-core, 4 shell, and 1 line_specific/cloud gene. Group 4 (71 genes) was highly conserved, with 68 core genes accompanied by only 2 shell and 1 line_specific/cloud gene. In contrast, Group 3 (63 genes) exhibited the highest enrichment of soft-core members (45 core, 14 soft-core, 3 shell, and 1 line_specific/cloud gene), whereas Group 1 (56 genes) contained a relatively higher proportion of dispensable genes (41 core, 7 soft-core, and 8 shell genes). Notably, Group 2 (53 genes) consisted exclusively of core genes, representing the most conserved clade within the family. Collectively, these results indicate that although the BnPYL family retains a highly conserved phylogenetic structure, individual clades exhibit varying degrees of gene gain and loss.

### Gene structure and conserved motif patterns of *BnPYLs*

**Figure 2:**
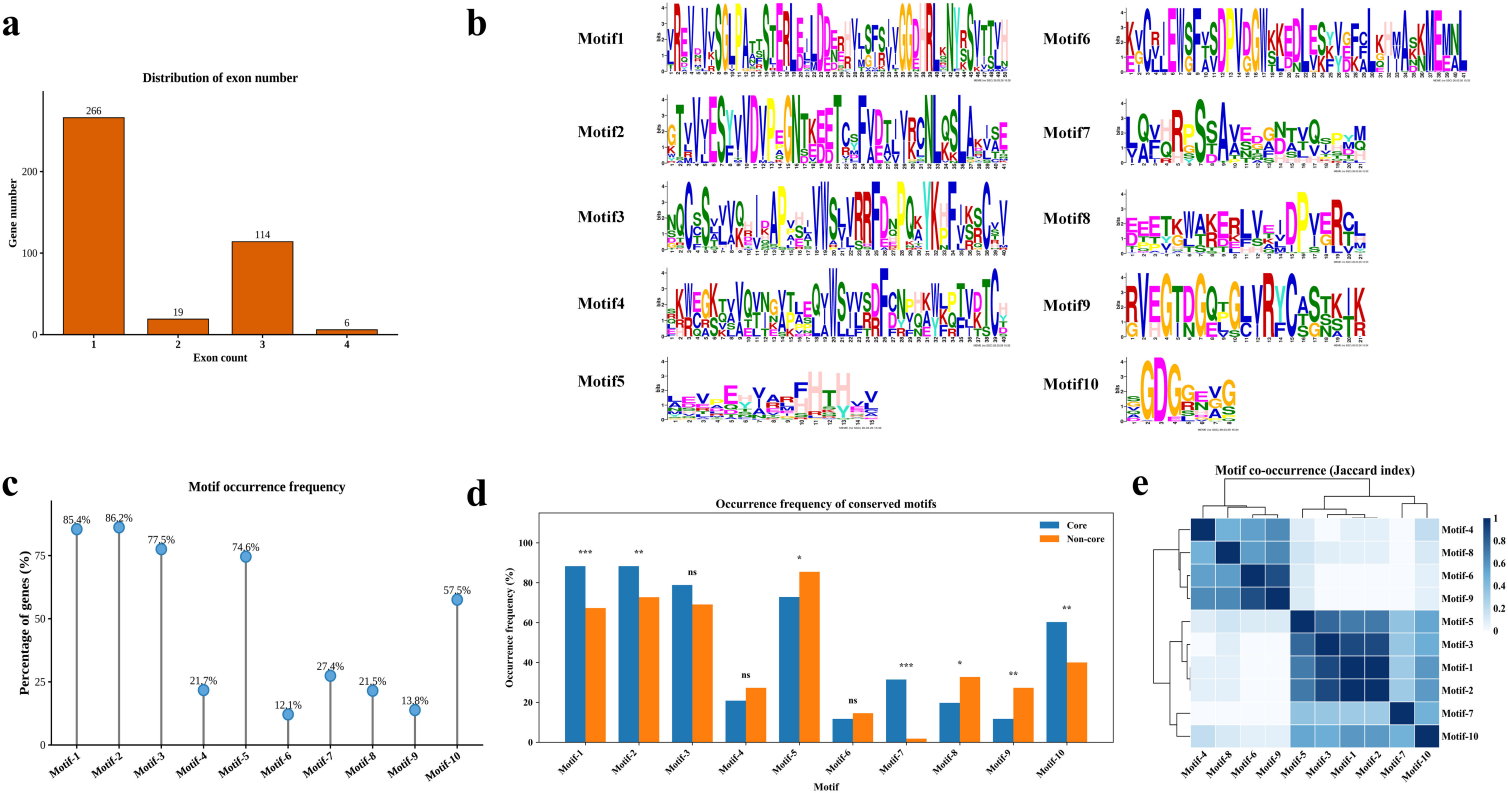
Exon–intron structure and conserved motif architecture of *BnPYLs*. **(A)** Frequency distribution of exon numbers in *BnPYLs* across the rapeseed pangenome. **(B)** Sequence logos of Motif 1–Motif 10 were generated from the full-length BnPYL protein sequences using MEME. The height of each amino acid indicates its relative conservation at that position, and the total height of each stack represents the information content. **(C)** Occurrence frequency of ten conserved motifs identified in *BnPYLs*. Percentages indicate the proportion of genes containing each motif. **(D)** Occurrence frequencies of Motif-1 to Motif-10 were compared between genes belonging to core orthogroups and non-core orthogroups. Statistical significance was evaluated using Fisher's exact test. Asterisks indicate significance levels: *P < 0.05, **P < 0.01, and *P < 0.001; ns indicates no significant difference. **(E)** Motif co-occurrence matrix based on the Jaccard index.

The exon–intron organization and conserved motif composition of the 405 PYL genes identified from the eight *Brassica napus* genomes revealed an overall pattern of strong structural conservation accompanied by limited diversification (**Figure 2A**; **Supplementary Table 4**). Most *BnPYLs* displayed a simple gene structure, with 266 genes (65.68%) containing a single exon and 114 genes (28.15%) containing three exons, whereas only 19 (4.69%) and 6 (1.48%) genes harbored two and four exons, respectively. This structural simplicity was mainly associated with the core component of the family. Among the 350 core genes, 233 were single-exon and 97 contained three exons, indicating that the conserved fraction of the family retains a highly compact architecture. By contrast, non-core genes showed relatively greater structural variation, and the three line_specific/cloud genes each exhibited distinct exon organizations.

A total of ten conserved motifs were identified in *BnPYLs* (**Supplementary Table 4, S5**). The identified motifs ranged from 8 to 50 amino acids in length (**Figure 2B**). In this study, these MEME-derived motifs refer to statistically conserved amino acid sequence patterns among BnPYL proteins, rather than direct annotations of known PYL functional domains. Among them, Motif-2 (86.2%), Motif-1 (85.4%), Motif-3 (77.5%), and Motif-5 (74.6%) were the most prevalent (**Figure 2C**), representing the major conserved motif framework of the family, whereas the remaining motifs occurred at lower frequencies.

To quantitatively evaluate motif conservation between core and non-core members, we compared the number of motifs per gene and the occurrence frequency of each motif (**Figure 2D**). Genes belonging to core orthogroups contained more motifs per gene than non-core members, with an average of 4.84 ± 1.01 motifs in core genes and 4.38 ± 0.91 motifs in non-core genes (Mann-Whitney U test, P = 0.0050). Among the dominant motifs, Motif-1 and Motif-2 were significantly more frequent in core genes than in non-core genes, with occurrence frequencies of 88.3% versus 67.3% for Motif-1 (Fisher’s exact test, P = 2.60 × 10^−4^) and 88.3% versus 72.7% for Motif-2 (P = 0.0051). Motif-10 was also enriched in core genes, occurring in 60.3% of core genes compared with 40.0% of non-core genes (P = 0.0053). In contrast, Motif-5, Motif-8, and Motif-9 were more frequent in non-core members. Consistent with the exon structure patterns, core genes showed higher motif conservation overall, particularly for the dominant motifs, while non-core genes displayed more variable motif compositions. Motif co-occurrence analysis further revealed a highly stable core module formed by Motif-1, Motif-2, and Motif-3, with strong pairwise associations between Motif-1/Motif-2 (Jaccard index = 0.991), Motif-2/Motif-3 (0.900), and Motif-1/Motif-3 (0.897) (**Figure 2E**). Together, these results indicate that the *B. napus* PYL family is dominated by structurally conserved core genes.

### Cis-regulatory features of *BnPYLs* in the pangenome

**Figure 3:**
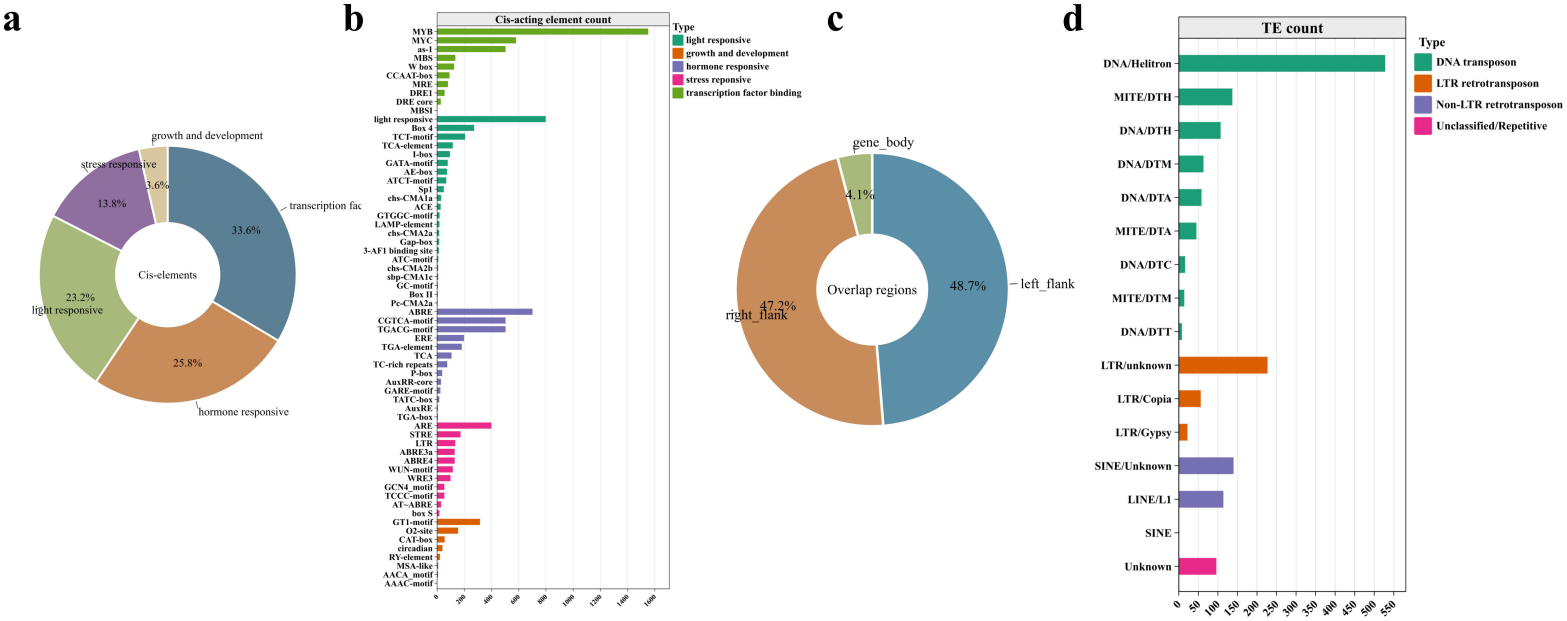
Promoter cis-elements and TE-associated variation of *Brassica napus* PYL genes. **(A)** Functional composition of cis-acting elements identified in BnPYL promoters. **(B)** Counts of individual cis-elements. **(C)** Genomic distribution of TE overlaps relative to BnPYL loci. **(D)** TE family composition of BnPYL-associated overlaps.

Promoter analysis across *BnPYLs* identified a total of 9,381 cis-acting elements, revealing a regulatory architecture strongly biased toward signal integration rather than basal developmental control. Among the five functional categories, transcription factor binding-related elements were the most abundant (3,148/9,381; 33.6%), followed by hormone-responsive elements (2,424; 25.8%) and light-responsive elements (2,172; 23.2%), whereas stress-responsive (1,299; 13.8%) and growth- and development-related elements (338; 3.6%) were comparatively underrepresented (**Figure 3A**; **Supplementary Table 6**). This distribution indicates that the promoter landscape of the rapeseed pangenome is dominated by combinatorial transcriptional regulation and environmental signal responsiveness, rather than by constitutive developmental motifs. At the level of individual motifs, MYB-binding sites were the most abundant single element (1,552), followed by light-responsive elements (798), ABRE (701), MYC (580), and the jasmonate-associated CGTCA-motif and TGACG-motif (both 503) (**Figure 3B**). Notably, MYB and MYC motifs together accounted for 67.7% of all transcription factor binding elements (2,132/3,148), suggesting that MYB/MYC-mediated regulatory motifs are highly represented in BnPYL promoters. In the light-responsive category, the canonical light-responsive element was the most frequent motif, together with substantial representation of Box 4 (272) and TCT-motif (206), suggesting that light-related regulatory elements are widely present in BnPYL promoters. By contrast, although growth- and development-related elements were globally scarce, GT1-motif (315) predominated within this class, suggesting that developmental regulation may rely on a limited number of key cis-regulatory modules rather than on broad motif redundancy.

### TE-associated diversification of *BnPYLs*

Across the *BnPYLs*, 1632 transposable element related overlaps were identified in gene associated regions, with a striking positional bias toward gene flanks. Of these, 795 events (48.7%) occurred in the left flank and 770 (47.2%) in the right flank, whereas only 67 (4.1%) mapped to the gene body (**Figure 3C**).

At the major TE class level, DNA transposons were the predominant category, accounting for 976 overlaps (59.8%), followed by LTR retrotransposons with 305 overlaps (18.7%), non-LTR retrotransposons with 255 overlaps (15.6%), and unclassified or repetitive elements with 96 overlaps (5.9%) (**Figure 3D**; **Supplementary Table 7**). Among individual TE subclasses, DNA/Helitron was the most abundant, with 528 overlaps (32.4%), indicating that Helitron-related DNA transposons are the major TE component associated with BnPYL loci. Other abundant subclasses included LTR/unknown with 227 overlaps (13.9%), SINE/Unknown with 140 overlaps (8.6%), MITE/DTH with 137 overlaps (8.4%), LINE/L1 with 114 overlaps (7.0%), and DNA/DTH with 107 overlaps (6.6%). The predominance of DNA transposons, particularly DNA/Helitron elements, suggests that DNA transposon activity has contributed substantially to sequence diversification around BnPYL loci. Although less abundant than DNA transposons, retrotransposon-derived elements were also widely represented, including LTR/unknown, LTR/Copia, LTR/Gypsy, LINE/L1, SINE/Unknown, and SINE elements. These insertions may influence local genome organization and regulatory context by altering nearby sequence composition, promoter structure, chromatin accessibility, or transcriptional activity.Taken together, these results support a model in which TEs have reshaped the pangenome primarily by accumulating around genes, where they may influence promoter context, local chromatin state, and gene expression divergence among accessions.

### Copy number conservation, structural variation, and duplication events analysis of *BnPYLs*

**Figure 4:**
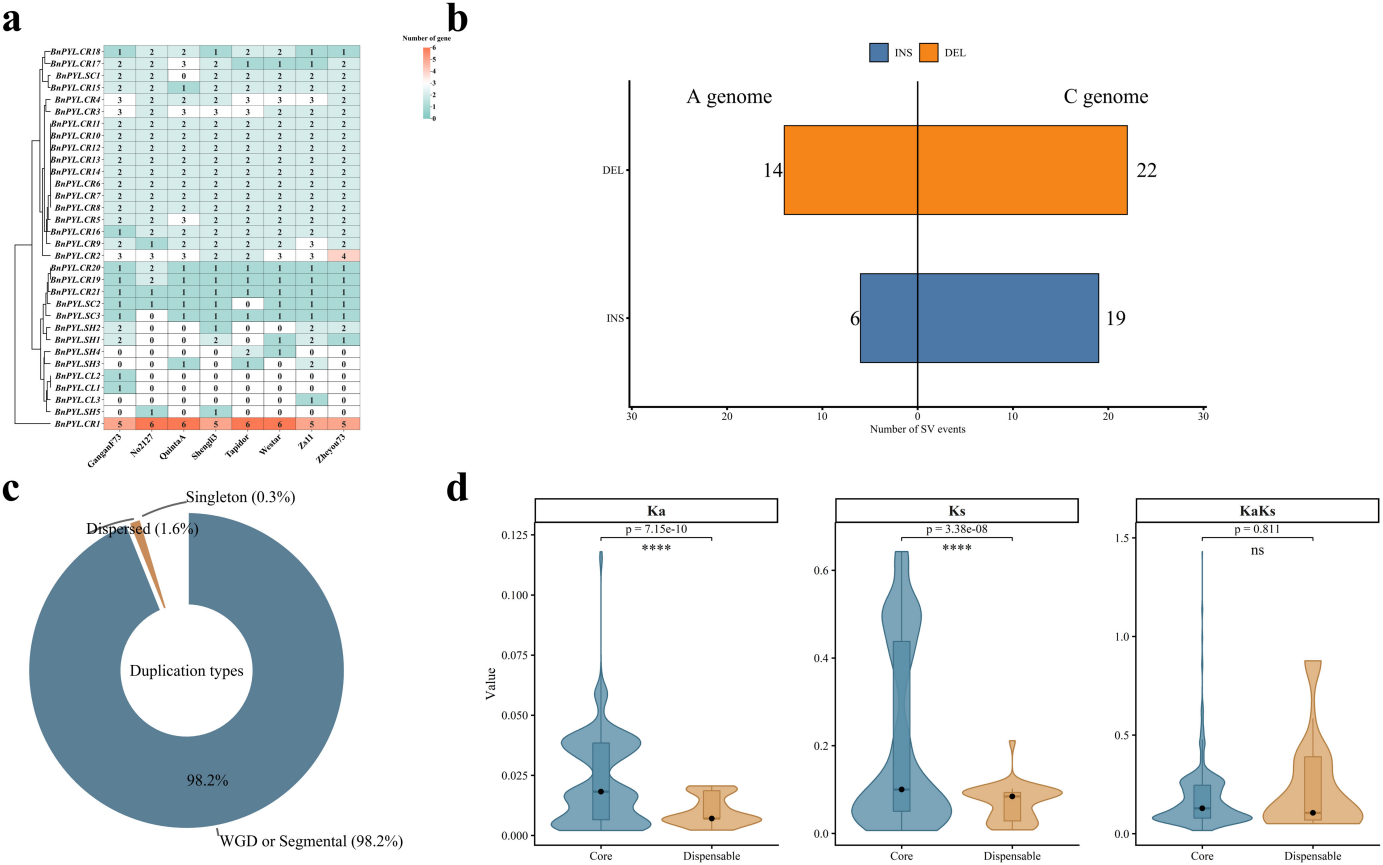
Copy number, structural variation, duplication type, substitution rate and synteny of *BnPYLs*. **(A)** Heatmap showing the copy number of *BnPYLs*. The numbers in the cells indicate gene copy number in each accession. **(B)** Numbers of insertion (INS) and deletion (DEL) events detected in the A and C subgenomes. Blue bars indicate insertions and orange bars indicate deletions. **(C)** Proportions of duplication types in *BnPYLs*, including whole-genome duplication or segmental duplication (WGD or Segmental), dispersed duplication, and singleton genes. **(D)** Violin plots showing the distributions of Ka, Ks, and Ka/Ks values for core and dispensable *BnPYLs*. Black dots indicate mean values. Statistical significance between core and dispensable groups was assessed separately for each metric using the Wilcoxon rank-sum test. Asterisks indicate significance levels: ****P < 0.0001.

Copy number variation, structural variation, and duplication analyses together indicate that the PYL gene family has a highly conserved core set but retains clear locus specific plasticity across the pangenome. In the CNV heatmap, most core genes were present at two copies in the majority of the eight accessions, pointing to strong conservation of the canonical ABA receptor repertoire (**Figure 4A**). Notably, *BnPYL.CR21* was the only single copy core gene detected consistently across all accessions, highlighting its exceptional evolutionary conservation within the pangenome. A subset of genes, however, showed marked copy number expansion or contraction. *BnPYL.CR1* was the most expanded locus, with five to six copies detected across all accessions, while *BnPYL.CR2* varied from two to four copies, indicating substantial accession specific dosage divergence. By contrast, several members in the BnPYL.SH and BnPYL.CL groups showed frequent copy loss, with some loci reduced to zero copies in multiple accessions, suggesting that copy number variation is concentrated in particular branches of the family rather than distributed evenly across all *BnPYLs*. Structural variation analysis further revealed a clear subgenome asymmetry. In the A genome, 14 deletions and 6 insertions were detected, whereas the C genome contained 22 deletions and 19 insertions (**Figure 4B**; **Supplementary Table 8**). The higher number of both insertion and deletion events in the C subgenome indicates that it has undergone more extensive structural remodeling than the A subgenome, which may underlie the stronger copy number variability observed at specific PYL loci. Duplication mode analysis showed that the expansion of the PYL family is overwhelmingly associated with large scale duplication. Whole genome duplication or segmental duplication accounted for 98.2% of all duplication events, whereas dispersed duplication represented only 1.6% and singleton genes only 0.3% (**Figure 4C**; **Supplementary Table 9**). Together, these results support a model in which the *BnPYLs* repertoire was established primarily through ancient genome-wide duplication and subsequently refined by uneven structural variation between subgenomes, especially within the C genome. This combination of deep duplication history and localized copy number reshaping is likely to have provided an important genomic basis for functional diversification of ABA signaling in the rapeseed pangenome.

### Purifying selection and conserved synteny highlight the evolutionary stability of *BnPYLs*

Comparative evolutionary analysis of *BnPYLs* revealed overall strong sequence conservation between core and dispensable groups, accompanied by measurable differences in substitution dynamics (**Figure 4D**). Both Ka and Ks distributions differed significantly between the two groups, with core genes exhibiting higher values than dispensable genes, whereas Ka/Ks ratios did not differ significantly. Despite these differences in absolute substitution rates, Ka/Ks values for both groups remained well below 1, indicating that both core and dispensable *BnPYLs* have evolved predominantly under purifying selection. This pattern suggests that dispensable genes are not subject to relaxed or positive selection but instead retain levels of functional constraint comparable to those of core genes, despite their lower synonymous and nonsynonymous substitution rates. Accordingly, the divergence between the two groups is better explained by differences in evolutionary rate rather than by shifts in the direction of selection.

**Figure 5:**
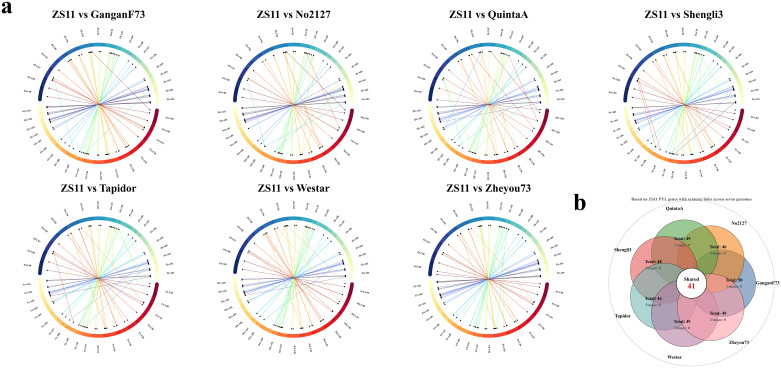
Syntenic relationships of PYL genes between ZS11 and seven Brassica napus genomes. **(A)** Pairwise Circos plots showing PYL collinear gene pairs between ZS11 and each of the seven genomes. Colored lines represent syntenic PYL gene pairs. **(B)** Venn-style summary showing the number of ZS11 PYL genes with syntenic links in each genome comparison. The central number indicates PYL genes shared across all seven comparisons.

Synteny analysis further showed that the PYL family is highly conserved across the rapeseed pangenome relative to the reference accession ZS11 (**Figures 5A, B**; **Supplementary Table 10**). Using ZS11 as the reference, 51, 47, 48, 50, 48, 49, and 49 ZS11 PYL loci had collinear PYL counterparts in GanganF73, No2127, QuintaA, Shengli3, Tapidor, Westar, and Zheyou73, respectively. The relatively narrow range in the number of collinear genes, from 47 to 51, indicates that the genomic positions of most BnPYL loci have remained stable across diverse accessions. Among these genomes, GanGan showed the highest number of collinear genes with ZS11, whereas No2127 showed the lowest, suggesting modest accession specific differences in local genomic rearrangements or gene retention. Taken together, these results indicate that *BnPYLs* combines strong positional conservation across genomes with consistent purifying selection at the sequence level, supporting the view that this ABA receptor family has remained evolutionarily constrained despite structural diversity at the pangenome level.

### Codon usage bias of *BnPYLs*

**Figure 6:**
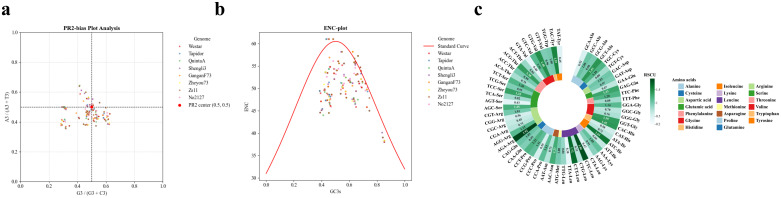
Codon usage features of *BnPYLs*. **(A)** PR2-bias plot of *BnPYLs*. Each point represents one gene. The dashed horizontal and vertical lines indicate the coordinates of the PR2 center at (0.5, 0.5). Different colors represent different genomes. **(B)** ENC-plot of *BnPYLs*. The red curve indicates the standard curve, and colored points represent individual genes from different genomes. **(C)** Circular plot showing the RSCU values of synonymous codons in *BnPYLs*. Codons are arranged around the circle, and the inner colored ring indicates the corresponding amino acid categories.

Codon usage analysis was performed as a supplementary sequence-level characterization of *BnPYLs* to evaluate synonymous codon bias and nucleotide compositional features within the gene family (**Supplementary Table 11**). In the PR2 plot, most genes clustered near the center of the graph, with A3/(A3 + T3) and G3/(G3 + C3) values mainly distributed around 0.5, indicating that the four nucleotides are used in a relatively balanced manner at the third codon position (**Figure 6A**). Moderate dispersion around the center, however, suggested that codon usage was not entirely random. The ENC–GC3s analysis further showed that most *BnPYLs* had moderate to high ENC values, mainly ranging from 45 to 60, indicating weak overall codon usage bias (**Figure 6B**).

The RSCU profile showed a mild preference for several A/U-ending codons, whereas some G/C-ending codons were used less frequently (**Figure 6C**; **Supplementary Table 12**). Importantly, the overall RSCU pattern was broadly similar across the seven genomes, indicating that codon usage bias in the PYL family is evolutionarily stable at the pangenome scale. Taken together, these results show that *BnPYLs* exhibit a weak but structured codon usage bias, in which mutation pressure provides the compositional background, while natural selection and other regulatory constraints also contribute to shaping synonymous codon choice.

### Tissue-specific expression patterns of *BnPYLs*

**Figure 7:**
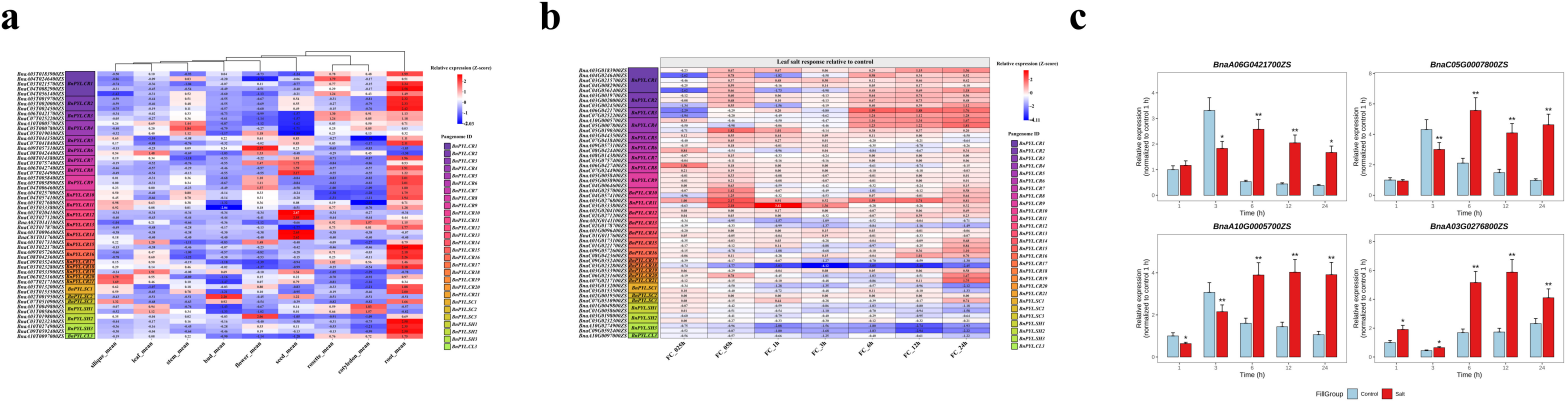
Expression profiles of *BnPYLs*. **(A)** Heatmap showing the relative expression levels of *BnPYLs* across nine tissues, including silique, leaf, stem, bud, flower, seed, rosette, cotyledon, and root. Gene names are shown on the left, and the color scale represents relative expression values (Z-score). **(B)** Heatmap showing the relative expression levels of *BnPYLs* under salt treatment at different time points. Columns represent 0.25 h, 0.5 h, 1 h, 3 h, 6 h, 12 h, and 24 h, and the color scale represents relative expression values (Z-score). **(C)** Bar plots showing the relative expression levels of four *BnPYLs* under control and salt treatment at 1, 3, 6, 12, and 24 h. Blue bars represent the control, and red bars represent salt treatment. Error bars indicate standard deviation.

Expression profiling across nine tissues, including bud, flower, leaf, seed, silique, root, cotyledon, rosette, and stem, revealed pronounced transcriptional divergence within the *BnPYLS*. A major feature of the dataset was the widespread enrichment of PYL transcripts in roots, where many members reached their highest expression levels. This root biased pattern was particularly evident for *BnPYL.SH3*, *BnPYL.CR10*, *BnPYL.CR2*, and *BnPYL.CR5*, and was also observed in several *BnPYL.CR1* copies, suggesting that ABA receptor mediated signaling is especially active in belowground tissues (**Figure 7A**).

In addition to this general root preference, several genes showed clear organ specific expression patterns. *BnPYL.CR4* was strongly expressed in flower and stem, indicating a likely role in the regulation of reproductive and aerial tissues. *BnPYL.CR11* showed relatively broad expression but was especially abundant in leaf and silique, whereas *BnPYL.CR5* accumulated strongly not only in root but also in bud, flower, seed, and silique, suggesting a broader contribution to both vegetative and reproductive development. *BnPYL.CR21* and *BnPYL.CR20* were also preferentially expressed in silique, supporting the idea that part of the PYL family may participate in fruit related developmental processes. In contrast, *BnPYL.CR3* showed notable expression in cotyledon and rosette, pointing to possible functions in early seedling establishment and vegetative growth. By comparison, several members, including *BnPYL.CR12*, *BnPYL.CR14*, *BnPYL.CR15*, *BnPYL.SC2*, and *BnPYL.SC3*, remained weakly expressed across nearly all sampled tissues, indicating that not all PYL genes contribute equally to basal ABA signaling under normal conditions. Importantly, even closely related paralogues often displayed distinct expression patterns, suggesting that substantial transcriptional divergence has occurred after gene duplication.

### Salt stress induces selective activation of *BnPYLs*

Salt stress transcriptome profiling across seven time points, including 0.25, 0.5, 1, 3, 6, 12, and 24 h, revealed substantial transcriptional divergence among *BnPYLs* when expression was measured as fold change relative to the control (**Figure 7B**). Overall, the heat map showed that most salt responsive *BnPYLs* were not activated immediately, but instead exhibited a more distinct transcriptional response at the middle and late stages of treatment. Several members of the BnPYL.CR subgroup showed sustained induction under salt stress, including *BnPYL.CR1*, *BnPYL.CR2*, *BnPYL.CR3*, *BnPYL.CR4* and *BnPYL.CR11*, whose transcript levels increased mainly after 6 h and remained elevated at 12 or 24 h. Particularly strong induction was observed for *BnaA10G0005700ZS* and *BnaA06G0421700ZS*, both of which shifted from weak or negative early responses to clear upregulation at later time points. *BnaC05G0007800ZS* also showed a pronounced late response, with induction becoming evident after 6 h, whereas *BnaA03G0276800ZS* exhibited a broader activation pattern and remained positively responsive across most treatment stages. In contrast, several genes, such as *BnPYL.CR13*, *BnPYL.CR18*, and members of the BnPYL.SH subgroup, especially *BnPYL.SH3*, were consistently downregulated, indicating that salt stress does not activate the entire PYL family uniformly but instead leads to clear functional partitioning among paralogues.

To further test the transcriptome based pattern, we selected four representative genes, *BnaA06G0421700ZS*, *BnaC05G0007800ZS*, *BnaA10G0005700ZS*, and *BnaA03G0276800ZS* (**Figure 7C**), for expression validation. These genes were chosen because they represented strong and sustained salt responsive candidates in the heat map, and the validation results supported their clear induction under salt treatment, particularly at later stages.

### Haplotype diversity in four salt responsive *BnPYLs* shows putative phenotypic differences under high salt–alkali conditions

**Figure 8:**
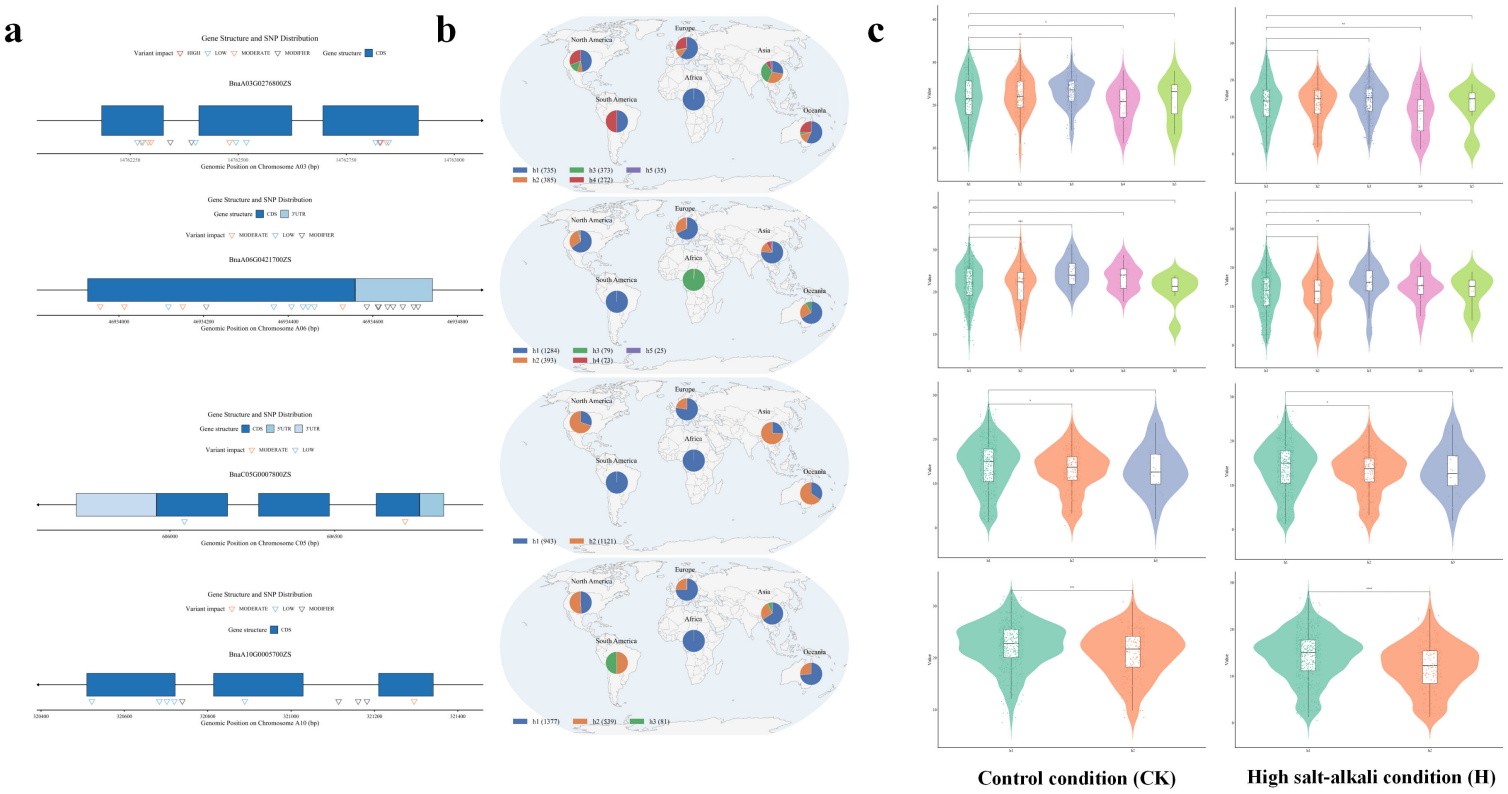
Gene structure, haplotype distribution and phenotypic variation of four salt-responsive *BnPYLs*. **(A)** Gene structures and SNP distributions of *BnaA03G0276800ZS*, *BnaA06G0421700ZS*, *BnaC05G0007800ZS*, and *BnaA10G0005700ZS*. Blue boxes indicate coding regions (CDS), light blue boxes indicate UTRs, and were further classified as low, moderate, or modifier impact variants according to predicted functional effects. **(B)** Geographic distributions of haplotypes for the four genes. Pie charts show the proportions of different haplotypes in accessions from different continents, and the number of accessions for each haplotype is indicated in the legend. These distribution maps were generated using the full set of genotyped accessions available for each gene, including 1,819 accessions for *BnaA03G0276800ZS*, 1,871 for *BnaA06G0421700ZS*, 2,082 for *BnaC05G0007800ZS*, and 2,016 for *BnaA10G0005700ZS*. **(C)** Violin plots showing trait values of different haplotypes under CK and high salt–alkali condition (H). Each panel corresponds to one gene, and each violin represents one haplotype group. Phenotypic association analysis was performed using the subset of 505 accessions with available trait data. Boxes indicate the interquartile range, center lines indicate median values, and points represent individual accessions. Statistical significance among haplotype groups was assessed using one-way ANOVA followed by Tukey’s honestly significant difference (HSD) *post hoc* test. P values for pairwise comparisons were adjusted using the Tukey HSD method. Asterisks indicate significant differences among haplotypes.

Haplotype analysis of the four salt responsive *BnPYLs* was conducted using 1,819 to 2,082 accessions, depending on genotype availability at each locus. Specifically, *BnaC05G0007800ZS*, *BnaA10G0005700ZS*, *BnaA06G0421700ZS*, and *BnaA03G0276800ZS* were resolved from 2,082, 2,016, 1,871, and 1,819 accessions, respectively. These four genes showed clear differences in allelic complexity. *BnaC05G0007800ZS* contained 2 SNPs and 2 haplotypes, *BnaA10G0005700ZS* contained 10 SNPs and 3 haplotypes, *BnaA03G0276800ZS* contained 15 SNPs and 5 haplotypes, and *BnaA06G0421700ZS* showed the highest sequence diversity, with 20 SNPs and 5 haplotypes (**Figure 8A**; **Supplementary Tables 13**, **S14**). Thus, the four loci differed markedly in both nucleotide variation and haplotype structure, ranging from a simple two haplotype pattern at *BnaC05G0007800ZS* to more complex allelic architectures at *BnaA06G0421700ZS* and *BnaA03G0276800ZS*.

This contrast was also reflected in haplotype frequency distributions. *BnaC05G0007800ZS* was dominated by two common haplotypes with comparable frequencies, indicating a relatively simple allelic structure. *BnaA10G0005700ZS* was represented mainly by one major haplotype, together with one secondary haplotype and one rare form. By contrast, *BnaA06G0421700ZS* and *BnaA03G0276800ZS* both contained five haplotypes, but the latter showed a more even distribution of haplotype frequencies, consistent with its greater sequence diversification. Geographic analysis further showed that the major haplotypes of all four genes were widely distributed across continents, whereas minor haplotypes tended to be regionally enriched, indicating that global sharing and local differentiation coexist within the natural population (**Figure 8B**).

To evaluate whether natural haplotype variation at these candidate loci was related to agronomic performance, we compared yield values among haplotype groups under the same environmental condition using the published field phenotypic dataset from Zhang et al. (2022). When these haplotypes were further compared for yield under both control and high salt alkali conditions, clear phenotypic differences emerged within each gene (**Figure 8C**). Significant yield variation among haplotypes was detected under at least one condition for all four loci. In genes with more complex haplotype structures, especially *BnaA06G0421700ZS* and *BnaA03G0276800ZS*, haplotype dependent yield differences were evident under both environments, suggesting stable phenotypic effects across conditions. The simpler loci, *BnaA10G0005700ZS* and *BnaC05G0007800ZS*, also showed significant haplotype associated differences, but these contrasts were concentrated among fewer major allelic classes. Importantly, haplotype-associated yield differences remained detectable under high salt–alkali stress, suggesting that natural variation in these four genes may be associated with yield potential under normal conditions and stress performance.

## Discussion

The *Brassica napus* PYL family shows a clear combination of evolutionary conservation and functional diversification. This pattern is consistent with the central role of PYR/PYL ABA receptors in plant stress signaling, in which ABA perception suppresses clade A PP2Cs and activates SnRK2 dependent downstream responses (Ma et al., 2009; Park et al., 2009). Because this pathway regulates core adaptive processes such as osmotic adjustment, stomatal regulation, and stress induced transcription, strong conservation of receptor genes would be expected. The predominance of core *BnPYLs* across the pangenome, together with Ka/Ks values consistently below 1 for both core and dispensable members, supports this view and indicates that most receptors have remained under strong purifying selection rather than undergoing substantial functional decay. Notably, although core *BnPYLs* exhibited higher absolute Ka and Ks values than dispensable *BnPYLs*, the absence of a significant difference in Ka/Ks ratios suggests that this pattern is unlikely to reflect relaxed functional constraint or positive selection in the core group. Instead, the elevated Ka and Ks values in core genes may be explained by differences in duplication age, whereby duplicated core gene pairs represent older duplication events and therefore have had more time to accumulate both synonymous and nonsynonymous substitutions (Qiao et al., 2019; Jiang et al., 2022). Similar conclusions have been drawn from *Arabidopsis* and other crops, where PYL receptors are generally conserved at the protein level even when their physiological outputs differ among paralogues (Santiago et al., 2009; Antoni et al., 2013; Kim et al., 2014).

At the same time, the present study indicates that conservation of coding function has not prevented extensive diversification in family composition and dosage. Almost all duplication events in the BnPYL family were attributable to whole genome or segmental duplication, whereas dispersed duplication made only a very small contribution. This pattern is in line with the polyploid origin of rapeseed and with the broader view that many regulatory gene families in B. napus were established on a duplicated genomic backbone and later refined by lineage specific gain, loss, and restructuring (Chalhoub et al., 2014; Song et al., 2020; Zhang et al., 2024). The strong retention of most *BnPYLs*, the narrow range of collinear genes among accessions, and the persistence of a single copy core locus such as *BnPYL.CR21* together suggest that dosage balance remains important for much of the family. By contrast, loci such as *BnPYL.CR1* and *BnPYL.CR2* appear to have retained greater copy number flexibility, implying that some receptor subgroups tolerate dosage shifts more readily than others. A similar combination of stable synteny and local structural plasticity has been described at the rapeseed pangenome scale, where conserved genomic organization coexists with substantial expression affecting variation among accessions (Verma et al., 2019; Mao et al., 2022). These observations suggest that the BnPYL family did not diversify mainly through recurrent gene birth, but through differential retention and remodeling after polyploidization.

The promoter and transposable element analyses further indicate that much of this diversification is regulatory rather than coding. *BnPYLs* promoters contained abundant MYB, MYC, ABRE, and light responsive elements, whereas TE associated variation was concentrated predominantly in gene flanking regions rather than gene bodies. This pattern is important because it suggests that receptor protein function is conserved, whereas the timing, intensity, and tissue specificity of expression remain more evolutionarily labile. The frequent occurrence of ABRE is biologically consistent with the role of ABA receptor genes, given the established role of AREB/ABF transcription factors in ABA dependent osmotic stress signaling (Fujita et al., 2005). The flanking bias of TE overlaps is also notable. Increasing evidence indicates that TEs often shape phenotypic diversity by altering promoter context, chromatin state, and transcriptional responsiveness rather than by directly disrupting coding regions (Stuart et al., 2016). In rapeseed and related Brassica species, structural variants and TE insertions are now recognized as major contributors to gene expression divergence and trait variation (Zhang et al., 2024). The present findings therefore support a model in which BnPYL diversification has been driven largely by promoter level rewiring superimposed on a conserved receptor framework.

This regulatory interpretation is further supported by the expression data. BnPYL transcription was clearly not uniform across organs. Instead, the family showed pronounced tissue partitioning, with many members reaching their highest transcript abundance in roots and others displaying organ biased accumulation in flower, stem, cotyledon, rosette, silique, or seed. Root enrichment is particularly meaningful because root centered ABA signaling is a well-established feature of this pathway. In *Arabidopsis*, *PYL8* has an especially important role in ABA signaling and the regulation of root growth, whereas other receptors contribute to partially overlapping but distinct functions in vegetative and reproductive tissues (Antoni et al., 2013; Zhao et al., 2016). The strong root biased expression of several rapeseed genes, including members of the CR and SH groups, suggests that root associated ABA perception has remained a major conserved function of the family across the pangenome. At the same time, the broad expression of some members and the narrow organ specificity of others indicate that duplicate retention has been accompanied by transcriptional partitioning. This type of divergence provides a plausible route by which a large receptor family can avoid complete redundancy while preserving a common biochemical mechanism. The weak but structured codon usage bias is consistent with this broader picture. Mutation pressure alone did not account for codon usage patterns, but the relatively high ENC values indicate that translational constraint remains moderate, which is consistent with a family under functional conservation but not strong sequence level innovation (Wright, 1990).

The salt stress response results provide particularly strong evidence for functional partitioning within the family. Salt treatment did not induce the entire BnPYL repertoire uniformly. Instead, transcriptional activation was concentrated in a subset of CR type receptors, especially *BnPYL.CR1*, *BnPYL.CR2*, *BnPYL.CR3*, *BnPYL.CR*4 and *BnPYL.CR10*, with four representative genes showing clear induction in both transcriptome and validation assays. This selective recruitment is consistent with earlier analyses of the rapeseed PYL family, which likewise identified only a subset of members as stress responsive (Di et al., 2018). It is also consistent with functional studies in *Arabidopsis*, rice, and wheat showing that enhanced activity of specific PYL receptors can increase abiotic stress tolerance, but that the phenotypic outcome depends strongly on receptor identity and expression pattern (Kim et al., 2014; Mao et al., 2022). This point is important because constitutive activation of ABA signaling can improve stress resistance but may also reduce growth or impose developmental costs (Kim et al., 2014). The delayed induction observed for several *BnPYLs* may therefore reflect a regulatory strategy that limits early growth penalties while allowing stronger ABA receptor activity during sustained stress. In this context, the late activation of *BnaA06G0421700ZS* and *BnaA10G0005700ZS*, together with the broader response of *BnaA03G0276800ZS*, appears especially relevant to saline environments in which prolonged, rather than immediate, stress adaptation is required.

The haplotype analysis adds a population genetic and agronomic dimension to this framework. The four salt-responsive genes differed markedly in SNP number and haplotype complexity, from the simple two-haplotype structure of *BnaC05G0007800ZS* to the much richer allelic architectures of *BnaA06G0421700ZS* and *BnaA03G0276800ZS*. More importantly, haplotypes at all four loci were associated with yield variation under both control and high salt-alkali conditions. This suggests that natural allelic variation in ABA receptors is not merely a molecular signature of population history, but has measurable effects on agronomic performance. Such a conclusion is consistent with recent evidence in wheat showing that modulation of the ABA receptor *TaPYL1-1B* improves drought tolerance and grain yield through increased water-use efficiency (Mao et al., 2022). It also helps reconcile two recurring observations in ABA receptor biology. On one hand, engineered elevation of receptor activity can enhance stress tolerance (Liang et al., 2017; Mega et al., 2019). On the other hand, excessive ABA signaling can compromise growth (Zhai et al., 2025). Naturally occurring haplotypes may offer a more balanced route, preserving adaptive value while avoiding the stronger penalties that sometimes accompany constitutive overexpression. The geographic distribution of major and minor haplotypes further suggests that both global sharing and regional differentiation have contributed to the current allelic landscape, which is consistent with local selection acting on a conserved pangenome background.

Taken together, these results support a three layer model for *BnPYLs* evolution in rapeseed. The first layer is a conserved receptor framework shaped by polyploid history and maintained by purifying selection. The second layer is a regulatory layer generated by promoter diversification, structural variation, and TE associated remodeling. The third layer is a population level layer in which a limited number of stress responsive loci retain agronomically meaningful haplotype variation. Under this model, most *BnPYLs* remain conserved because ABA perception is indispensable, whereas a smaller subset functions as flexible tuning points for tissue specificity, stress timing, and environmental adaptation. This interpretation provides a useful foundation for future work. In particular, *BnaA06G0421700ZS*, *BnaC05G0007800ZS*, *BnaA10G0005700ZS*, and *BnaA03G0276800ZS* emerge as strong candidates for allele level validation, promoter dissection, and marker assisted selection aimed at improving salt alkali adaptation and yield stability in rapeseed. Future functional studies, including gene editing, overexpression, and complementation assays, will be necessary to confirm their causal roles in salt–alkali adaptation and yield stability in rapeseed.

## Data Availability

The original contributions presented in the study are included in the article/**Supplementary Material**. Further inquiries can be directed to the corresponding authors.
